# Resveratrol reduces inflammation-related Prostate Fibrosis

**DOI:** 10.7150/ijms.44443

**Published:** 2020-07-19

**Authors:** Enzo Vicari, Alessandro Arancio, Vito Emanuele Catania, Beatrice Ornella Vicari, Giuseppe Sidoti, Roberto Castiglione, Michele Malaguarnera

**Affiliations:** 1 Department of Clinical and Experimental Medicine, University of Catania, 95123 Catania, Italy.; 2 Department of Medical, Surgical Sciences and Advanced Technologies “G.F. Ingrassia”, University of Catania, 95123 Catania, Italy.; 3 Research Centre "The Great Senescence", University of Catania, 95126 Catania, Italy.; 4 UOSD Medicina Interna Ambulatorio Andrologia & Endocrinologia ARNAS-Garibaldi, 95123 Catania, Italy.

**Keywords:** Prostate fibrosis, small prostate volume, Meares and Stamey test, LUTS, IPSS, NIH-CPSI, Resveratrol

## Abstract

Inflammation-related prostate fibrosis (PF) is strongly associated with impaired urethral function and lower urinary tract symptoms (LUTS) severity. The aim of this study was to investigate the effects of RSV in patients with small prostate volume and LUTS. Sixty-four patients with PF were randomized either to RSV therapy (group A= 32 patients) or placebo (group B= 32 patients). At baseline (T0) and after 2-months (T2), patients of both groups underwent administration of NIH-Chronic Prostatic Symptom Index (NIH-CPSI) and International Prostate Symptom Score (IPSS) questionnaires for prostatitis and LUTS, respectively, and Expressed Prostatic Secretion (EPS) assays. After two months, only, group A patients treated with RSV showed significant symptomatic improvement of all NIH-CPSI and IPSS subscale scores, as well as a better EPS assay after prostate massage, in terms of high amount of prostatic volume and reduced white blood cells counts. Our data suggested pharmacological advantage after 2-month treatment with RSV in selected patients with PF for the treatment of voiding and storage complaints.

## Introduction

Prostatic inflammation has been suggested to be a major etiological factor for benign prostatic hyper-plasia (BPH) and lower urinary tract symptoms (LUTS). End stage of inflammatory diseases results in fibrosis, characterized by the excessive deposition of collagen. Growing evidence shows that the degree of inflammation is correlated with symptoms severity and disease progression of BPH (nondependent prostate volume), indicating the heterogeneity of BPH disease 1.

Studies in animal and human support the hypothesis that the development of fibrosis could be a reversible process when the cause is removed or suppressed in various tissues, including kidneys or liver 2,3.

Resveratrol (RSV) is a nutraceutical belonging to stilbenoid group, widely distributed in the plant kingdom and with several therapeutic effects. RSV is found in grapes, nuts, berries, and various other plants. Aside the various dietary sources, the RSV supplements also contain the cis- and trans-isomers of this phenolic compound, although trans-RSV is the principal form found. The trans-form shows greater stability and more biological activity than the cis-form. RSV *in vivo* is characterized by rapid metabolism and low bioavailability.

This molecule has been mainly studied as a complementary therapy against cardiovascular and brain disorder 4-7.

Apart from its cardiologic and neuroprotective affects, RSV also exerts anticancerogenic, antiviral, anti-inflammatory and antioxidant properties 8. This last two characteristics allow RSV to be a potential treatment of chronic pathologies in aging related diseases.

Previous experimental studies identified that RSV can improve the outcome of prostatitis, since RSV treatment represses and reverses prostate fibroblast to myofibroblast phenoconversion *in vitro* 9,10.

Albeit *in vitro* studies and animal models, as well as the pre-clinical evidence have demonstrated the anti-inflammatory effects of RSV, the clinical results are not as promising as the pre-clinical data and substantial conflicting data exist 11-14.

The majority of clinical studies have been designed and conducted in order to evaluate diverse metabolic outcomes. Moreover, approximately half of the published studies find an anti-inflammatory effect, and among these, some find an improvement of insulin sensitivity, whereas the other half of studies are unable to detect any anti-inflammatory effect of RSV.

Prostate fibrosis (PF) represents a particular inflammatory condition of chronic prostatitis (CP) strongly associated with impaired urethral function through fibrotic changes in periurethral prostatic tissues 12, 13.

*In vitro* and *in vivo* studies have addressed to RSV promising effects on the pathogenetic mechanisms of fibrosis not only via inflammation but also via the mast cells suppression and interfering to TGF-β/Wnt/β-catenin pathway 14, 15.

Therefore, the aim of this study was to investigate the therapeutic effect of RSV in patients with small prostate volume and LUTS.

## Materials and Methods

### Subjects

This observational study was conducted at the Andrology and Endocrinology Unit Clinic, Policlinic University of Catania (Catania, Italy), between February 2017 and July 2018. Sixty-four selected male outpatients (median age: 35 years, range: 30-49 years) with a diagnosis of chronic inflammatory prostatitis (NIH type IIIa) and suggestive multiple symptoms and signs of prostatic fibrosis were enrolled in this study.

The diagnosis of NIH type IIIa variant fibrotic was made 6-12 months before the patients were included in this study.

All patients and controls underwent collection of their clinical history, administration of NIH-Chronic Prostatitic Symptom Index (NIH-CPSI) and International Prostate Symptom Score (IPSS) questionnaires for prostatitis and LUTS, respectively, and a physical examination 16.

The clinical characteristics of patients are described in the **Table l**.

### Inclusion criteria for diagnostic symptoms

Since it is well known that when inflammation mainly periurethral in the prostate evolved in fibrosis, this leads the progression of urinary dysfunction, for the purpose of this study, we adopted the following diagnostic criteria:

- ***Clinical documented history*** of recurrent urinary tract infections (UTI) who may be asymptomatic between episodes or may present chronic genitourinary pain for more than 3 months according to the European Association of Urology (EAU) guidelines 17;
- ***Validated questionnaire for IPSS*** National Institutes of Health Chronic Prostatitis Symptom Index (NIH-CPSI), a self-reported questionnaire widely used to assess Chronic Prostatitis (CP/CPSI) patients and IPSS for diagnosis of LUTS. A total NIH-CPSI score ≥8 was considered indicative of prostatitis, at baseline;
- LUTS encompasses a wide range of symptoms, categorized by the International Continence Society (ICS) as storage, voiding, and post-micturition. The severity of LUTS was assessed using the IPSS, Each of the questions is rated from 0 (not at all) to 5 (almost always), and according to the total symptom score, the severity of LUTS can be graded as mild (0-7), moderate (8-19) or severe (20-35), since a total IPSS ≥8 points was considered to indicate the presence of LUTS 18;
- Proven chronic inflammatory prostatitis (NIH type IIIa), was diagnosed when no infection was detected through urine culture, sperm culture or from the expressed prostate secretion (EPS) after prostatic massage 21.

A patient was assigned to the NIH category IIIa group if at the Meares-Stamey four-glass test, the white blood cell (WBC) count in the EPS was equal to or greater than 10 per high power field (HPF) or when the WBC count in the voided 5-10 ml urine after prostatic massage (postmassage voided urine - VB3 fraction of the Stamey test) was equal to or greater than 5 per HPF 20,21.

- Abnormal signs at physical examination and transrectal ultrasound (TRUS) suggestive of fibro-sclerotic prostatitis, as previously reported 22. At the end of this study, we arbitrarily chose patients with small prostate volume (<30 ml) who displayed 2 or more abnormalities for each lobe of the prostate, such as multiple areas of hypoechogenicity (associated with edema) and hyperechogenicity (associated with areas of calcification), without additional ultrasound abnormal signs at the seminal vesicles. In the present study, only the larger prostatic calculi (with the diameter over 3 mm) with more echogenic foci, that caused acoustic shadowing, were considered significant prostatic calcification. This last criterion has been adopted according to Park et al. and colleagues 20, who reported that prostatic inflammatory changes were closely associated with this type of calcification. Instead, hyperechoic areas without shadowing and tiny stippled calcifications (<3 mm in largest diameter) were not considered as prostatic calculi for the purpose of this analysis.
- Completely absent or low expressed prostate secretion after prostate massage at the Meares-Stamey four-glass test, performed during the diagnostic work-up of prostatitis 23.

### Exclusion criteria for diagnostic symptoms

Exclusion criteria were as follows

- Conditions that were potential LUTS inducing factors, such as: patients aged >50 years in order to exclude the LUTS of benign prostatic hyperplasia (BPH); clinically apparent bladder or prostate cancer; neurologic disease that could influence voiding symptoms; uncontrolled diabetes mellitus; history of a previous lower urinary tract surgery; history of radiotherapy to the pelvis;
- History of chronic bacterial prostatitis (NIH type II) with a positive bacteriological finding at sperm culture or at the Meares-Stamey four-glass test;
- Subjects suffering from chronic or acute illness that could interfere with the study, who were taking medications that could interfere in the study, such as: anti-inflammatory drugs, proton pump inhibitors (PPIs), antidepressants, and antispasmodic agents, and who consumed antibiotics in the four weeks prior the study;
- Obesity. Defined as a body mass index (BMI) greater than or equal to 30 kg/m^2^);
- Subjects affected by major concomitant diseases, with known anatomical abnormalities of the urinary tract or with evidence of other urological diseases, and with residual urine volume of >100 mL resulting from bladder outlet obstruction;
- Patients with a history of prostatitis treatments.

### Outcome measures and methods

#### Primary outcome measures

The primary outcome of this study were the changes in the NIH-CPSI total score and IPSS total score, both measured at baseline (T0) and on the end of 2^nd^ month (T2).

#### Secondary outcome measures

The secondary outcome included:

- The assessment at T0 and T2 of change in the subtotal NIH-CPSI, including symptoms such as pain or discomfort, urination, impact of symptom, and the subscale about Quality of life (QOL), and subtotal IPPS/QOL scores, subcategorized into: voiding (items 1, 3, 5, 6), storage (items 2, 4, 7) and postmicturition symptoms;
- Changes in EPS assays obtained after prostatic massage.

### Treatment

Patients in both groups of prostatic fibrosis were allocated at random into two subsets which were assigned either to the treatment with RSV 19.8 (group A=32), or placebo in tablet (group B=32) both one tablet twice daily for two consecutive months.

Randomization was achieved using a computer program. The first random set of numbers was assigned to treatment, including together 32 patients with inflammatory fibrotic prostatitis (intervention group). The second random set of 32 patients with inflammatory fibrotic prostatitis received placebo (control group).

Study subjects were informed of the risks and requirements of the study, and the study protocol was approved by the internal Institutional Review Board and informed written consent was obtained from each patient.

### Statistical analyses

Statistical analysis was performed with SPSS ver. 12.0 (SPSS Inc., Chicago, IL, USA).

Quantitative data were expressed as median and range, and qualitative data were expressed as percentages throughout the study. Intragroup differences in NIH-CPSI or IPSS questionnaire scores before/after therapy were analysed using Wilcoxon's signed rank test. Mann-Whitney U tests, or the chi-square test or Fisher's exact test were used for analyses that compared different groups. A statistically significant difference was accepted when p value was lower than 0.05.

### Results

The demographic and baseline characteristics of the variables studied in both patient groups are shown in **Table 1**. There was no significant difference between the two groups of total 64 patients, eligible for the comparative study analysis: treatment with RSV (intervention group or group A) or placebo (control group or group B) (**Table 1**).

### Comparison between RSV treated with placebo treated in primary outcomes

In the treated group we observed that the total NIH-CPSI score were reduced from 17.5 to 14.0 and the total IPSS score from 19.5 to 12.5, respectively before vs after treatment. The total NIH-CPSI score and the total IPSS score in placebo group did not show significant modifications after the treatment. The comparison between RSV treated and placebo showed a significant reduction in NHI-CIPSI score and in total IPSS score (**Table 2**).

Both groups with prostate fibrosis at T0 showed a high percentage of patients with completely absent or low amount of prostatic secretion after prostate massage (Stamey test), with significantly high WBC counts per high power field in VB3, corroborating more likely the diagnosis of inflammatory prostatitis (NIH, category IIIA) (**Table 2**). At T2, after treatment and prostate massage, the group A had a better EPS, in terms of increased amount of prostate volume secreted and WBC counts per high power field. Significantly lower than those found in matched group in the pretreatment (**Table 2**).

Before the treatment (T0), total NIH-CPSI scores did not show any significant difference between the two groups. After 2 months of treatment (T2), the total score changed significantly in both groups (*p* < 0.05). In the treated group, the total NIH-CPSI score reduced from 17.5 (range 14-22) to 14.0 (range 9-19) but was unmodified in the placebo group (**Table 2**).

In RSV treated group, we observed a significant reduction in pain subscale, in urinary subscale and in QoL subscale (subtotal NIH-CPSI score) and in storage IPSS, in voiding IPSS and in QoL subscale (subtotal IPSS score) comparing before vs. after treatment. No significant modifications were shown in placebo treated group after the treatment. The comparison between Treated vs. Placebo groups showed a significant difference in both the subtotal NIH-CPSI score and subtotal IPSS score (**Table 2**). Hence, although no significant difference existed between the two groups at baseline in all NIH-CPSI subscale score, at T2 significant decreases of pain, urination, and quality of life were shown in the RSV group (group A), whereas these same subscales scores were unchanged in the placebo group (group B) (**Table 2**). Regarding obstructive (LUTS) symptoms, IPSS score showed no significant difference between the groups A and B at baseline (T0). At T2, compared with baseline symptoms significant decreases were observed in the total IPSS total score, IPSS-storage, IPSS-voiding, IPSS-QOL, in patients of group A (intervention group), but were unmodified in the placebo group.

## Discussion

The results of this trial show that the treatment with RSV decreased PF and LUTS, increasing the quality of life. In fact, we observed a decrease 3,5 points in total NIH-CPSI score, 7,0 points in total IPSS score and the increase of 46.9% in EPS after prostate massage.

PF is a consequence of chronic inflammation, causing hyperplasia of cells with a smooth muscle cell phenotype and altering the turnover of collagen and other extracellular matrix components.

Agents that prevent or slow the proliferation of smooth muscle cells and decrease excessive collagen deposition may provide new therapeutic strategies or be used as supplement to the current immune-modulatory therapy of fibrostenosing 24.

The decrease of the primary outcomes is associated with a reduction of secondary outcomes: 1.6 points pain, 1.7 points urinary subscale, 1.5 points storage IPSS, 4.5 points voiding IPSS (**Table 2**).

Chronic inflammatory prostatitis (NIH, cat IIIA) (CP), a common disease in urology, has been suggested to contribute to the aetiology of lower urinary tract symptoms (LUTS) by inducing fibrotic changes in periurethral prostatic tissues and promote urethral stiffness and LUTS 25.

In clinical practice, approximately half of LUTS patients have a relatively small (30 mL or less) prostate volume (small PV), indicating the heterogeneity of this disease 26. Therefore, the classical dynamic and static components of prostate cannot explain the exact pathophysiology of small PV- LUTS 27, 28. Thus, prostatic inflammation-induced fibrosis has gained increasing attention as a major contributing factor in the pathogenesis of small prostate volume and LUTS. In these patients, the co-presence of multiple prostatic calcifications having larger prostatic calculi with the diameter over 3 mm has a pathological connection with prostatic inflammatory changes and fibrosis 29,30. In particular, inflammation of the periurethral area, can contribute, via an excess of collagen deposition and fibrosis in the prostate, to impair urethral function, with urethral stiffness which in turn maximizes induction of LUTS 31-32.

In a previous study, we described three diagnostic categories (prostatitis, prostate-vesiculitis and prostate-vesiculo-epididymitis), distinguishing into two major variants, with group-specific ecostructural findings called hypertrophic-congestive, and fibro-sclerotic. In the present study we enrolled patients who responded precisely to diagnostic criteria of prostatitis inflammation-induced fibrosis. The present study investigated changes related to a 2-months treatment with RSV in patients with prostate fibrosis, through the analysis of irritative and obstructive symptoms as well as measures of expressed prostate secretion assays obtained after prostatic massage. Here, the intervention group of patients treated with RSV showed significant symptomatic improvement of all NIH-CPSI and IPSS subscale scores, as well as a better EPS assay after prostate massage, in terms of high amount of prostatic volume and reduced white blood cells counts.

RSV has been shown to reduce inflammation to inhibit fibrosis in cultured vascular smooth muscle cells in rat model as well as to inhibit intimal hyperplasia in an *in vivo* model of vascular injury 33-35.

RSV tested in an animal model of chronic prostatitis resversed mast cell activation, decreased collagen content, the maximum capacity of the bladder, residual urine volume, maximum voiding pressure and the expression levels of α‑SMA, a marker of fibrosis. It is possible that these effects have been due to the decreased activity of TGF‑β/Wnt/β‑catenin signaling, involved in the mast cell activation and fibrosis process, and SCF/c-Kit signaling pathway, related to the proliferation and differentiation of cells 36, 37. The inactivation of these pathways seems caused by the activation of SIRT1 pathway as a result of RSV administration 38.

Pyo et al. (2017) found similar decreased histopathological changes in prostate when mice with estradiol-induced prostatitis were treated with RSV 39.

One of main problem of RSV formulation is the low bioavailability in humans 40.

The absorption of the dietary relevant 25 mg oral dose was at least 70 %, with peak plasma levels of RSV, metabolites of 491± 90 ng/ml and a plasma half-life of 9.2±0.6 hours. Most of the oral dose was recovered in urine. Although the systemic bioavailability of RSV is low, accumulation of RSV and its potentially active metabolite may still produce tumour preventive and other effects 41, 42.

The differences between the results in clinical trials on different pathological conditions may be attributable to differences in the study populations. Genders, age, dosage of RSV, length of the study, and the health status of the participants have been suggested to be factors that may influence the results. Especially, a large difference has been found in the concentration and dosage of RSV among the clinical studies. No consensus exists on which concentration of RSV may be ideal in relation to anti-inflammatory effects.

In our study RSV plays an important role to reduce the chronic inflammation that promote excessive collagen deposition in the prostate and is at least part reversible 43.

## Conclusions

Currently no therapeutic agents have shown a specifically curative activity on prostatic fibrosis.

Although to the best of our knowledge, no reports have investigated the effects of RSV on reversibility of fibrosis in the prostate.

The results of the present study suggest that RSV may be considered as a potential target for the treatment of LUTS in patients with small prostate volume and prostate fibrosis, through a reduction of the progression urinary dysfunction.

## Figures and Tables

**Table 1:** Clinical characteristics and variables in men with prostate fibrosis before the treatment (group A) or placebo (group B)

	Group A (n=32)	Group B (=32)	P value
Age (years)	35 (30-49)	35 (30-49)	ns
Prostate volume (ml)	28.4 (22-30)	27.1 (22-30)	ns
PSA (ng/mL)	2.0 (1.3-2.5)	2.0 (1.4-2.4)	ns
PVR (ml)	49 (5-70)	45 (5-65)	ns
Total IPSS score	19.5 (9-24)	19.0 (9-24)	ns
No. of patients with severe symptoms (overall score >20) (%)	11 (34.3%)	10 (31.2%)	ns
Total NIH-CPSI score	17.5 (14-22)	18.0 (14-23)	ns
**Periurethral prostatic calcification (diameter >3 mm) at TRUS**			
Mean number (range)	3 (2-4)	3 (2-4)	ns
Mean diameter (mm) (range)	7 (4-12)	6.5 (4-11)	ns
WBC on EPS after prostate massage or VB3 (counts/hpf)	11 (8-16)	10 (8-15)	ns

Values are presented as mean±standard deviation. ns = not significant difference; **Abbreviations:** PSA: serum prostate specific antigen; PVR: postvoid residual urine, IPSS: International Prostate Symptom Score. IPSS: international prostate symptom score; hpf: high power field; NIH-CPSI: National Institutes of Health Chronic Prostatitis Symptom Index; TRUS: Transrectal ultrasonography; EPS: expressed prostatic secretion; WBC: white blood cell.

**Table 2:** Outcome measures analysis as a function of time of the treatment (group A) or placebo (group B) with RSV in studied patients with prostate fibrosis. Values were expressed as mean and range of percentages (in parentheses)

	PROSTATE FIBROSIS			
	Group A (intervention group)		Group B (placebo group)	
Study time-point	T0 (n=32)	T2 (n=32)	T0 (n=32)	T2 (n=32)
**Primary outcome**				
***Symptoms***				
Total NIH-CPSI score	17.5 (14-22)	14.0*° (9-19)	18.0 (14-23)	19.5 (13-24)
Total IPSS score	19.5 (9-24)	12.5*° (7-22)	19.0 (9-24)	19.0 (12-24)
No. with severe (IPPS score >20) symptoms	5 (15.6%)	4 (12.5%)	6 (18.7%)	7 (21.8%)
***Signs***				
EPS: volume (ml) after prostate massage	0.05 (0-0.1)	0.3*° (0-0.45)	0.05 (0-0.1)	0.05 (0-0.1)
No. patients without EPS after prostate massage	28 (87.5%)	13 (40.6%)*°	29 (90.6%)	29 (90.6%)
WBC on EPS after prostate massage or VB3	11 (8-16)	6*° (5-9)	10 (8-15)	12 (9-16)
**Secondary outcome**				
***Subtotal NIH-CPSI score***				
Pain subscale	9.4 (8-14)	7.8*° (4-10)	10.8 (8-14)	10.7 (7-13)
Urinary subscale	5.0 (3-6)	3.3*° (1-4)	5.8 (3-7)	4.0 (2-5)
Quality-of-Life (QoL) subscale	3.7 (3-5)	2.7*° (2-4)	4.2 (3-6)	3.8 (2-6)
***Subtotal IPSS score***				
Storage IPSS (items 2,4,7)	7.0 (5-12)	5.5*° (3-9)	7.0 (5-12)	7.0 (6-11)
Voiding IPSS (items 1, 3, 5 and 6)	9.5 (8-20)	5.0*° (4-12)	9.0 (8.0-20)	9.5 (8-19)
QoL subscale	3.0 (2.0-4)	2.0*° (1.4-3)	3.0 (2.0-4)	3.0 (2.0-4.0)

T0= before the treatment; T2= 2 months afterward; *p<0.05 intragroup comparison, Group A T2 vs. Group A T0. °*p*<0.05 intergroup comparison, Group A T2 vs. Group B T2.
